# 2-Methyl­benzene-1,3-di­ammonium dinitrate

**DOI:** 10.1107/S1600536814001561

**Published:** 2014-01-25

**Authors:** Dhouha Ben Hassen, Walid Rekik, Houcine Naïli, Tadeusz Lis, Roman Grobelny

**Affiliations:** a Laboratoire de Physico-Chimie de l’Etat Solide, Département de Chimie, Faculté des Sciences de Sfax, BP 802, 3018 Sfax, Tunisia; b Institute of Low Temperature and Structure Research, Polish Academy of Sciences, 2 Okolna, 50-422 Wroclaw, Poland; c Faculty of Chemistry, University of Wroclaw, Joliot-Curie 14, 50-383, Wroclaw, Poland

## Abstract

In the crystal structure of the title salt, C_7_H_12_N_2_^2+^·2NO_3_^−^, the nitrate ions are located in the vicinity of the protonated amine groups, accepting strong N—H⋯O hydrogen bonds. Each ammonium group is involved in a total of three such inter­actions with neighbouring nitrate ions, generating a three-dimensional network. In addition, there are π–π inter­actions between the aromatic rings of centrosymmetrically related di­ammonium moieties, with a centroid–centroid distance of 3.682 (1) Å.

## Related literature   

For applications of amine salts, see: Jayaraman *et al.* (2002 ▶). For hydrogen bonding, see: Steiner (2002 ▶). For related structures, see: Garza Rodríguez *et al.* (2013 ▶); Gao & Ng (2012 ▶); Riahi *et al.* (2012 ▶). For comparable crystal packing arrangements, see: Abrahams *et al.* (2013 ▶); Glidewell *et al.* (2004 ▶).
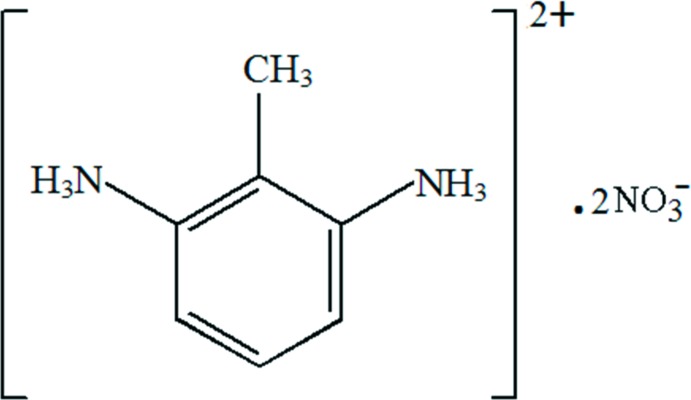


## Experimental   

### 

#### Crystal data  

C_7_H_12_N_2_^2+^·2NO_3_^−^
*M* *_r_* = 248.21
Monoclinic, 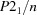

*a* = 10.494 (3) Å
*b* = 7.417 (2) Å
*c* = 13.487 (4) Å
β = 91.46 (5)°
*V* = 1049.4 (5) Å^3^
*Z* = 4
Mo *K*α radiation
μ = 0.14 mm^−1^
*T* = 293 K
0.36 × 0.30 × 0.16 mm

#### Data collection  

Nonius KappaCCD diffractometer
Absorption correction: analytical (de Meulenaer & Tompa, 1965 ▶) *T*_min_ = 0.708, *T*_max_ = 0.982
11810 measured reflections
2400 independent reflections
1617 reflections with *I* > 2σ(*I*)
*R*_int_ = 0.000

#### Refinement  

*R*[*F* ^2^ > 2σ(*F* ^2^)] = 0.038
*wR*(*F* ^2^) = 0.091
*S* = 0.84
2400 reflections
179 parameters
H atoms treated by a mixture of independent and constrained refinement
Δρ_max_ = 0.28 e Å^−3^
Δρ_min_ = −0.23 e Å^−3^

### 

Data collection: *COLLECT* (Nonius, 1998 ▶); cell refinement: *SCALEPACK* (Otwinowski & Minor, 1997 ▶); data reduction: *DENZO* (Otwinowski & Minor, 1997 ▶) and *SCALEPACK*; program(s) used to solve structure: *SHELXS97* (Sheldrick, 2008 ▶); program(s) used to refine structure: *SHELXL97* (Sheldrick, 2008 ▶); molecular graphics: *DIAMOND* (Brandenburg & Berndt, 1999 ▶); software used to prepare material for publication: *WinGX* (Farrugia, 2012 ▶).

## Supplementary Material

Crystal structure: contains datablock(s) global, I. DOI: 10.1107/S1600536814001561/lr2121sup1.cif

Structure factors: contains datablock(s) I. DOI: 10.1107/S1600536814001561/lr2121Isup2.hkl

CCDC reference:

Additional supporting information:  crystallographic information; 3D view; checkCIF report

## Figures and Tables

**Table 1:** Hydrogen-bond geometry (Å, °)

*D*—H⋯*A*	*D*—H	H⋯*A*	*D*⋯*A*	*D*—H⋯*A*
N1—H11⋯O5	0.91 (2)	1.85 (2)	2.746 (2)	168 (2)
N1—H12⋯O1^i^	0.93 (2)	1.93 (2)	2.845 (2)	168 (2)
N1—H13⋯O3^ii^	0.85 (2)	2.07 (2)	2.891 (2)	161 (2)
N2—H21⋯O5^i^	0.94 (2)	1.91 (2)	2.808 (2)	161 (2)
N2—H22⋯O1	0.89 (2)	1.96 (2)	2.839 (2)	172 (2)
N2—H23⋯O2^iii^	0.91 (2)	2.05 (3)	2.958 (2)	174 (2)

Symmetry codes: (i) 
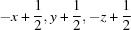
; (ii) 
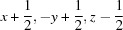
; (iii) 
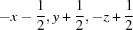
.

**Fig. 1.**
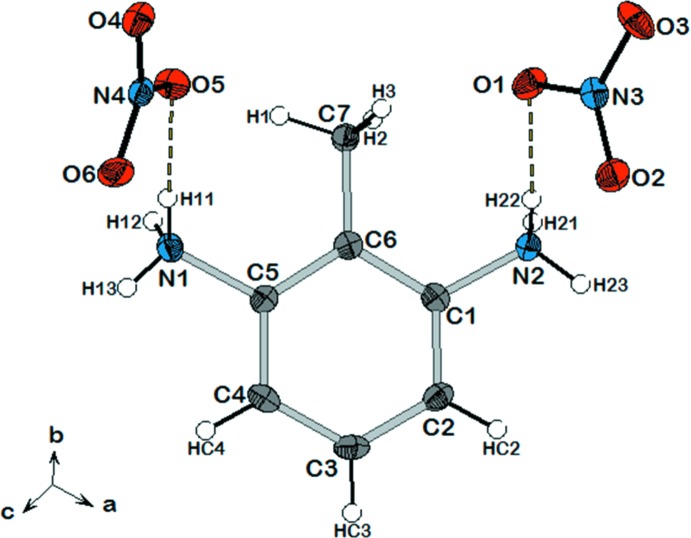
Asymmetric unit of the title salt. Displacement ellipsoids for non-H atoms are presented at the 50% probability level.

**Fig. 2.**
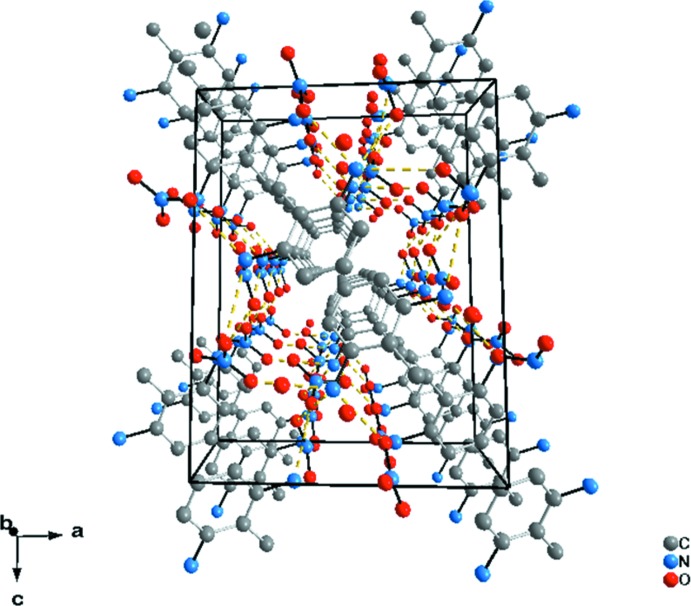
A perspective view of the crytal structure of (I). Hydrogen atoms are omitted for clarity.

**Fig. 3.**
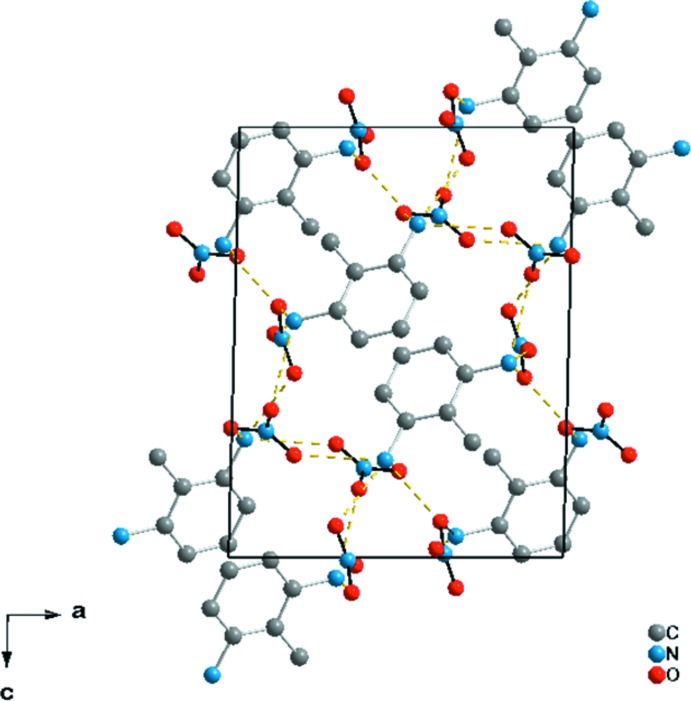
Projection of the structure of (I) along the b axis. Hydrogen atoms are omitted for clarity

## supplementary crystallographic information

### 1.1. Synthesis and crystallization

The title compound is resulting from a chemical reaction between three reagents: 2,6-di­amino­toluene (C_7_H_10_N_2_), nitric acid HNO_3_ and nitrate zinc hexahydrate Zn(NO_3_)_2_·6H_2_O. In the molar ratio 1:2:1, 0.12 g. The amine was dissolved in a little amount of distilled water, then 0.12 g of nitric acid and 0.29 g of zinc nitrate were added. The mixture was stirred for 15 minutes and the clear solution is allowed to stand at room temperature. The slow evaporation gives rise to the formation of dark brown crystals. The X-ray analysis investigation proves that a the divalent metal is not part of the structure and that the obtained phase is (C_7_H_12_N_2_). 2(NO_3_)

### 1.2. Refinement

Crystal data, data collection and structure refinement details are summarized in Table 1. H atoms bonded to N were found in a difference map and freely refined while H bonded to carbon atom were positioned geometrically and allowed to ride on their parent atom, with C—H = 0.93 Å, and *U*_iso_ = 1.2*U*_eq_(C) for aromatic and C—H = 0.96 Å, *U*_iso_ = 1.5*U*_eq_(C) for methyl.

### 2. Results and discussion

The amine salt family of compounds have attracted more attention due to their potential importance (Jayaraman *et al.*, 2002; Steiner *et al.*, 2002). In this paper, we report the synthesis and the crystal structure of a new amine nitrate salt (C_7_H_12_N_2_)^2+^ 2(NO_3_)^-^, (I). The asymmetric unit of (I), represented in figure 1, contains one protonated di­amine and two nitrate anions with general positions for all atoms. Both protonated nitro­gen atoms of organic cation are engaged in hydrogen bonds with two crystallographic independent nitrate groups. So that, nitro­gen atoms play the role of donor and oxygen atoms are acceptors in the model of hydrogen bond. Within these inter­molecular hydrogen bonds, the D···A distances vary from 2.745 (2) to 2.960 (2) Å. Within the nitrate groups, the N—O distances and the O—N—O angles are in the normal ranges (Garza Rodríguez *et al.*, 2013; Riahi *et al.*, 2012; Gao & Ng, 2012). Indeed, the N—O bond lengths range from 1.235 (2) to 1.284 (2) Å and the O—N—O bond angles are between 118.42 (16) and 122.46 (17) °. These values show that each nitrate anion exhibits a slightly distorted C_3h_ geometry. Within the aromatic rings of the organic moiety, the C—C and C—C—C angles are in the ranges 1.384 (3)—1.403 (3) Å and 115.68 (18)—123.15 (17) °. These values are comparable with those seen in other amine salts where the used amine contains a similar aromatic system (Riahi *et al.*, 2012; Gao *et al.*, 2012). The shortest inter­centroid distance between two aromatic systems is equal to 3.682 (1) Å. This value proves the existence of p···p inter­actions which contribute to the crystal stability. Comparable inter­centroid distances and inter­planar spacing between two parallel aromatic rings, have already been observed in the literature (Abrahams *et al.*, 2013; Glidewell *et al.* 2004). A perspective view of the structure shows an anionic stacking and a cationic one along the crystallographic *b* axis (figure 2). The obtained anionic and cationic layers, which are parallel to (-1 0 1) plane and inter­linked by N—H···O bonds, alternate along [1 0 - 1] direction (figure 3).

### Crystal data

**Table:**

C_7_H_12_N_2_^2+^·2NO_3_^−^	*F*(000) = 520
*M**_r_* = 248.21	*D*_x_ = 1.571 Mg m^−^^3^
Monoclinic, *P*2_1_/*n*	Mo *K*α radiation, λ = 0.71073 Å
Hall symbol: -P 2yn	Cell parameters from 11810 reflections
*a* = 10.494 (3) Å	θ = 3.1–27.5°
*b* = 7.417 (2) Å	µ = 0.14 mm^−^^1^
*c* = 13.487 (4) Å	*T* = 293 K
β = 91.46 (5)°	Prism, brown
*V* = 1049.4 (5) Å^3^	0.36 × 0.30 × 0.16 mm
*Z* = 4	

### Data collection

**Table:**

Nonius KappaCCD diffractometer	2400 independent reflections
Radiation source: fine-focus sealed tube	1617 reflections with *I* > 2σ(*I*)
Horizontally mounted graphite crystal monochromator	*R*_int_ = 0.000
Detector resolution: 9 pixels mm^-1^	θ_max_ = 27.5°, θ_min_ = 3.1°
CCD rotation images, thick slices scans	*h* = −13→13
Absorption correction: analytical (de Meulenaer & Tompa, 1965)	*k* = 0→9
*T*_min_ = 0.708, *T*_max_ = 0.982	*l* = 0→17
11810 measured reflections	

### Refinement

**Table:**

Refinement on *F*^2^	Primary atom site location: structure-invariant direct methods
Least-squares matrix: full	Secondary atom site location: difference Fourier map
*R*[*F*^2^ > 2σ(*F*^2^)] = 0.038	Hydrogen site location: inferred from neighbouring sites
*wR*(*F*^2^) = 0.091	H atoms treated by a mixture of independent and constrained refinement
*S* = 0.84	*w* = 1/[σ^2^(*F*_o_^2^) + (0.057*P*)^2^] where *P* = (*F*_o_^2^ + 2*F*_c_^2^)/3
2400 reflections	(Δ/σ)_max_ < 0.001
179 parameters	Δρ_max_ = 0.28 e Å^−^^3^
0 restraints	Δρ_min_ = −0.23 e Å^−^^3^

### Special details

**Table:**

Geometry. All e.s.d.'s (except the e.s.d. in the dihedral angle between two l.s. planes) are estimated using the full covariance matrix. The cell e.s.d.'s are taken into account individually in the estimation of e.s.d.'s in distances, angles and torsion angles; correlations between e.s.d.'s in cell parameters are only used when they are defined by crystal symmetry. An approximate (isotropic) treatment of cell e.s.d.'s is used for estimating e.s.d.'s involving l.s. planes.
Refinement. Refinement of *F*^2^ against ALL reflections. The weighted *R*-factor *wR* and goodness of fit *S* are based on *F*^2^, conventional *R*-factors *R* are based on *F*, with *F* set to zero for negative *F*^2^. The threshold expression of *F*^2^ > σ(*F*^2^) is used only for calculating *R*-factors(gt) *etc*. and is not relevant to the choice of reflections for refinement. *R*-factors based on *F*^2^ are statistically about twice as large as those based on *F*, and *R*- factors based on ALL data will be even larger.

### Fractional atomic coordinates and isotropic or equivalent isotropic displacement parameters (Å^2^)

**Table:**

	*x*	*y*	*z*	*U*_iso_*/*U*_eq_	
N1	0.32257 (14)	0.5382 (2)	0.04883 (13)	0.0161 (3)	
H11	0.330 (2)	0.416 (3)	0.0529 (16)	0.034 (6)*	
H12	0.3819 (19)	0.590 (3)	0.0923 (16)	0.029 (6)*	
H13	0.338 (2)	0.573 (3)	−0.0094 (18)	0.033 (6)*	
N2	−0.03954 (14)	0.5320 (2)	0.27455 (11)	0.0154 (3)	
H21	0.0007 (18)	0.582 (3)	0.3308 (15)	0.021 (5)*	
H22	−0.0346 (18)	0.413 (3)	0.2809 (14)	0.023 (5)*	
H23	−0.124 (2)	0.558 (3)	0.2749 (15)	0.031 (5)*	
C1	0.02007 (15)	0.5914 (2)	0.18309 (12)	0.0143 (3)	
C2	−0.04968 (15)	0.7016 (2)	0.11933 (13)	0.0174 (4)	
HC2	−0.1311	0.7390	0.1357	0.021*	
C3	0.00273 (15)	0.7559 (2)	0.03071 (13)	0.0189 (4)	
HC3	−0.0441	0.8288	−0.0129	0.023*	
C4	0.12492 (15)	0.7017 (2)	0.00712 (13)	0.0173 (4)	
HC4	0.1606	0.7373	−0.0522	0.021*	
C5	0.19263 (14)	0.5937 (2)	0.07346 (12)	0.0142 (3)	
C6	0.14394 (15)	0.5344 (2)	0.16332 (12)	0.0135 (3)	
C7	0.21965 (15)	0.4140 (2)	0.23249 (12)	0.0160 (3)	
H1	0.3076	0.4499	0.2331	0.024*	
H2	0.2124	0.2913	0.2103	0.024*	
H3	0.1873	0.4240	0.2982	0.024*	
O1	0.00268 (10)	0.15667 (16)	0.30012 (10)	0.0216 (3)	
O2	−0.18762 (11)	0.13185 (17)	0.23748 (9)	0.0237 (3)	
O3	−0.11592 (13)	−0.06969 (16)	0.34182 (9)	0.0260 (3)	
N3	−0.10232 (13)	0.07181 (18)	0.29321 (11)	0.0171 (3)	
O4	0.37995 (10)	−0.08942 (15)	0.01918 (9)	0.0212 (3)	
O5	0.37031 (11)	0.17901 (15)	0.08249 (9)	0.0203 (3)	
O6	0.31797 (11)	0.13625 (18)	−0.07227 (9)	0.0257 (3)	
N4	0.35592 (12)	0.07296 (19)	0.00802 (10)	0.0158 (3)	

### Atomic displacement parameters (Å^2^)

**Table:**

	*U*^11^	*U*^22^	*U*^33^	*U*^12^	*U*^13^	*U*^23^
N1	0.0172 (7)	0.0150 (8)	0.0161 (8)	−0.0007 (6)	0.0037 (6)	0.0003 (6)
N2	0.0128 (7)	0.0164 (8)	0.0170 (8)	0.0001 (6)	0.0014 (6)	0.0007 (6)
C1	0.0162 (7)	0.0126 (7)	0.0141 (8)	−0.0036 (6)	−0.0002 (6)	−0.0014 (7)
C2	0.0130 (7)	0.0157 (8)	0.0234 (10)	0.0005 (6)	−0.0016 (7)	0.0001 (7)
C3	0.0192 (8)	0.0159 (8)	0.0213 (10)	0.0000 (6)	−0.0063 (7)	0.0031 (7)
C4	0.0225 (9)	0.0149 (8)	0.0143 (9)	−0.0036 (6)	−0.0013 (7)	0.0012 (7)
C5	0.0132 (7)	0.0118 (8)	0.0176 (9)	−0.0012 (6)	−0.0010 (6)	−0.0026 (7)
C6	0.0153 (8)	0.0098 (7)	0.0154 (9)	−0.0025 (6)	−0.0023 (6)	−0.0025 (6)
C7	0.0150 (7)	0.0155 (8)	0.0176 (9)	0.0008 (6)	0.0019 (6)	0.0007 (7)
O1	0.0135 (6)	0.0183 (6)	0.0329 (7)	−0.0023 (5)	−0.0030 (5)	0.0016 (5)
O2	0.0155 (6)	0.0332 (7)	0.0223 (7)	−0.0006 (5)	−0.0038 (5)	0.0015 (6)
O3	0.0429 (8)	0.0146 (6)	0.0209 (7)	−0.0057 (6)	0.0086 (6)	0.0019 (5)
N3	0.0184 (7)	0.0156 (7)	0.0172 (7)	−0.0013 (6)	0.0025 (6)	−0.0027 (6)
O4	0.0195 (6)	0.0116 (6)	0.0327 (7)	−0.0002 (5)	0.0041 (5)	−0.0021 (5)
O5	0.0292 (7)	0.0166 (6)	0.0151 (6)	0.0035 (5)	0.0002 (5)	−0.0037 (5)
O6	0.0246 (7)	0.0360 (8)	0.0163 (7)	0.0093 (6)	−0.0041 (5)	0.0017 (6)
N4	0.0124 (6)	0.0170 (7)	0.0181 (8)	0.0005 (5)	0.0014 (5)	−0.0009 (6)

### Geometric parameters (Å, º)

**Table:**

N1—C5	1.470 (2)	C4—C5	1.384 (2)
N1—H11	0.91 (2)	C4—HC4	0.9300
N1—H12	0.93 (2)	C5—C6	1.398 (2)
N1—H13	0.85 (2)	C6—C7	1.504 (2)
N2—C1	1.465 (2)	C7—H1	0.9600
N2—H21	0.94 (2)	C7—H2	0.9600
N2—H22	0.89 (2)	C7—H3	0.9600
N2—H23	0.91 (2)	O1—N3	1.2703 (17)
C1—C2	1.382 (2)	O2—N3	1.2371 (19)
C1—C6	1.399 (2)	O3—N3	1.2475 (19)
C2—C3	1.388 (2)	O4—N4	1.2388 (18)
C2—HC2	0.9300	O5—N4	1.2817 (18)
C3—C4	1.389 (2)	O6—N4	1.2367 (18)
C3—HC3	0.9300		
			
C5—N1—H11	110.0 (13)	C5—C4—C3	118.78 (15)
C5—N1—H12	110.8 (12)	C5—C4—HC4	120.6
H11—N1—H12	108.5 (19)	C3—C4—HC4	120.6
C5—N1—H13	109.0 (15)	C4—C5—C6	123.34 (14)
H11—N1—H13	110 (2)	C4—C5—N1	118.67 (15)
H12—N1—H13	108.4 (19)	C6—C5—N1	117.99 (15)
C1—N2—H21	111.6 (11)	C5—C6—C1	115.58 (15)
C1—N2—H22	110.8 (13)	C5—C6—C7	121.71 (14)
H21—N2—H22	107.1 (18)	C1—C6—C7	122.69 (14)
C1—N2—H23	112.2 (13)	C6—C7—H1	109.5
H21—N2—H23	109.3 (17)	C6—C7—H2	109.5
H22—N2—H23	105.5 (18)	H1—C7—H2	109.5
C2—C1—C6	122.68 (15)	C6—C7—H3	109.5
C2—C1—N2	118.10 (14)	H1—C7—H3	109.5
C6—C1—N2	119.22 (15)	H2—C7—H3	109.5
C1—C2—C3	119.49 (15)	O2—N3—O3	122.10 (14)
C1—C2—HC2	120.3	O2—N3—O1	118.62 (14)
C3—C2—HC2	120.3	O3—N3—O1	119.27 (14)
C2—C3—C4	120.11 (16)	O6—N4—O4	122.38 (14)
C2—C3—HC3	119.9	O6—N4—O5	118.82 (14)
C4—C3—HC3	119.9	O4—N4—O5	118.80 (14)
			
C6—C1—C2—C3	1.5 (3)	N1—C5—C6—C1	179.86 (14)
N2—C1—C2—C3	−178.15 (15)	C4—C5—C6—C7	178.77 (15)
C1—C2—C3—C4	−0.8 (3)	N1—C5—C6—C7	−1.4 (2)
C2—C3—C4—C5	−0.2 (2)	C2—C1—C6—C5	−1.1 (2)
C3—C4—C5—C6	0.6 (2)	N2—C1—C6—C5	178.57 (14)
C3—C4—C5—N1	−179.28 (15)	C2—C1—C6—C7	−179.79 (15)
C4—C5—C6—C1	0.0 (2)	N2—C1—C6—C7	−0.2 (2)

### Hydrogen-bond geometry (Å, º)

**Table:**

*D*—H···*A*	*D*—H	H···*A*	*D*···*A*	*D*—H···*A*
N1—H11···O5	0.91 (2)	1.85 (2)	2.746 (2)	168 (2)
N1—H12···O1^i^	0.93 (2)	1.93 (2)	2.845 (2)	168 (2)
N1—H13···O3^ii^	0.85 (2)	2.07 (2)	2.891 (2)	161 (2)
N2—H21···O5^i^	0.94 (2)	1.91 (2)	2.808 (2)	161 (2)
N2—H22···O1	0.89 (2)	1.96 (2)	2.839 (2)	172 (2)
N2—H23···O2^iii^	0.91 (2)	2.05 (3)	2.958 (2)	174 (2)

Symmetry codes: (i) −*x*+1/2, *y*+1/2, −*z*+1/2; (ii) *x*+1/2, −*y*+1/2, *z*−1/2; (iii) −*x*−1/2, *y*+1/2, −*z*+1/2.
